# Mineralized Microgels via Electrohydrodynamic Atomization: Optimization and In Vitro Model for Dentin–Pulp Complex

**DOI:** 10.3390/gels9110846

**Published:** 2023-10-25

**Authors:** Iriczalli Cruz-Maya, Rosaria Altobelli, Marco Antonio Alvarez-Perez, Vincenzo Guarino

**Affiliations:** 1 Institute of Polymers, Composites and Biomaterials (IPCB), National Research Council of Italy, Mostra d’Oltremare Pad. 20, Viale J.F. Kennedy 54, 80125 Naples, Italy; 2 Tissue Bioengineering Laboratory of DEPeI-FO, Universidad Nacional Autonoma de Mexico (UNAM), Mexico City 04510, Mexico; marcoalv@unam.mx

**Keywords:** atomization, composite hydrogels, pulpar cells, dental tissue engineering

## Abstract

There is growing interest in the use of micro-sized hydrogels, including bioactive signals, as efficient platforms for tissue regeneration because they are able to mimic cell niche structure and selected functionalities. Herein, it is proposed to optimize bioactive composite microgels via electrohydrodynamic atomization (EHDA) to regenerate the dentin–pulp complex. The addition of disodium phosphate (Na_2_HPO_4_) salts as mineral precursors triggered an in situ reaction with divalent ions in solution, thus promoting the encapsulation of different amounts of apatite-like phases. Morphological analysis via image analysis of optical images confirmed a narrow distribution of perfectly rounded particles, with an average diameter ranging from 223 ± 18 μm to 502 ± 64 μm as a function of mineral content and process parameters used. FTIR, TEM, and EDAX analyses confirmed the formation of calcium phosphates with a characteristic Ca/P ratio close to 1.67 and a needle-like crystal shape. In vitro studies—using dental pulp stem cells (DPSCs) in crown sections of natural teeth slices—showed an increase in cell viability until 14 days, recording a decay of proliferation at 21 days, independent on the mineral amount, suggesting that differentiation is started, as confirmed by the increase of ALP activity at 14 days. In this view, mineralized microgels could be successfully used to support in vitro osteogenesis, working as an interesting model to study dental tissue regeneration.

## 1. Introduction

The dentin–pulp complex is a composite tissue formed by dental pulp and dentin. The former is a soft tissue embedding tissue-specific cells (i.e., pulp cells, odontoblasts, immune system cells, neurons, and endothelial cells) into an intricate network composed of extracellular matrixes and blood vessels. It is responsible for the nutrition of surrounding tissues (i.e., pulp), immune protection, and the formation of dentin, a calcified tissue that surrounds the pulp, forming the pulp cavity [1]. The dentin–pulp complex function is to maintain the structural integrity and normal function of teeth that can be affected by infection (i.e., caries, periodontitis) or trauma, leading to the need for root canal treatment. In this context, the most invasive problems involve the loss of dental pulp, usually related to problems of fragility, morphological changes, and root pain [2].

A valid alternative solution to the current endodontic treatments is the tissue engineering approach that is focused on the use of bio-inspired materials to fabricate a three-dimensional scaffold, able to reproduce the native microenvironment for cells in terms of chemical mechanical strength and able to support and guide the regeneration process of forming tissues [3,4,5].

In the last two decades, a large variety of biopolymers with recognized biological functions has been investigated to support specific cell interaction mechanisms (i.e., adhesion, proliferation, and differentiation) as a function of their peculiar chemical and/or morphological signals [6,7,8]. Among them, the use of materials with hydrogel-like behavior can offer a great added value for dental applications to form a three-dimensional (3D) network with a tunable porous microstructure, high hydrophilicity, and optimal transport properties, particularly useful for nutrient supply and a targeted diffusion of bioactive molecules [7,9,10,11]. Similar approaches have been recently used in place of calcium phosphate granules to investigate the odontogenic differentiation and DPSCs growth in 3D structures to support the dentin–pulp complex regeneration [12,13,14].

In this view, the downsizing of hydrogels to the micrometric scale, i.e., microgels, can provide a versatile tool to confine morphological, biophysical, and biochemical signals into a niche-like area to more efficiently control the interactions of pulp cells with the surrounding matrix, as a function of the relative composition, bioactive signal loading, and biomechanical properties. Moreover, the use of microgels can be suitable to facilitate the filling of irregular bone defects in order to replace the morphological/structural properties of the native tissue. Moreover, peculiar features of microgels in terms of high specific surface area, in comparison with commercial powder and granules, may positively contribute to supporting biocompatibility at the interface. Accordingly, in vitro studies have recently confirmed that microgels with tailored composition and microstructure can positively address in vitro cell response, supporting adhesion and proliferation and triggering differentiation mechanisms in an osteogenic way [15,16,17].

Electrohydrodynamic atomization (EHDA) identifies a group of high throughput and low-cost technologies based on the application of high voltage to manipulate polymer solutions in the form of particles from hundreds down to tens of microns in size, with highly tunable properties in terms of size, morphology, and composition [18]. Different from conventional electrospray technology, the droplets are collected into a crosslinking bath (i.e., CaCl_2_) onto the collector, enabling the formation of stable particles with a rounded shape and homogeneous size distribution prior to reaching the excess of charge into the droplet surface, deputed to the formation of particles with sub-micrometric/nanometric size [19].

The use of EHDA can satisfy the increasing interest in the microfabrication of substrates able to mimic the environmental conditions of natural tissues by chance to combine different materials (i.e., bioactive proteins, mineral precursors) with proven bio-recognition properties and efficient biological response in vitro and/or in vivo [20]. Herein, a new approach is proposed based on EHDA for the fabrication of bioactive composite microgels able to support the in vitro response of dental pulp stem cells (DPSCs) for the regeneration of mineralized tissues such as the dentin–pulp complex.

Sodium alginate (SA) has been selected to form the 3D structure due to its recognized biocompatibility and stability using electrical forces [21]. Indeed, SA is characterized by selected patterns of molecular sequences—in particular α-L-guluronic acid (G units), ionically reactive with divalent cations (i.e., Ca^2+^, Cu^2+^, Mg^2+^, Sr^2+^)—able to promote the formation of ionic inter-chain bridges that are crucial to stabilize the droplet shape and structure during the EHDA deposition [22,23,24]. Moreover, the ionotropic crosslinking capability of SA may allow the integration of different bioactive molecules (i.e., proteins, drugs, and inorganic compounds) to improve the properties of SA-based materials [25]. Therefore, in this work, SA-based microgels were prepared by the EHDA process and the use of salts in solution to trigger an in situ reaction with divalent ions in order to encapsulate mineral phases with apatite features during the atomization process. An accurate characterization to evaluate the chemical/physical properties of composite as a function of the mineral content has been performed. Furthermore, selected composite microgels were investigated in vitro with DPSCs to validate their use for the regeneration of the dentin–pulp complex.

## 2. Results and Discussion

**Figure 1:**
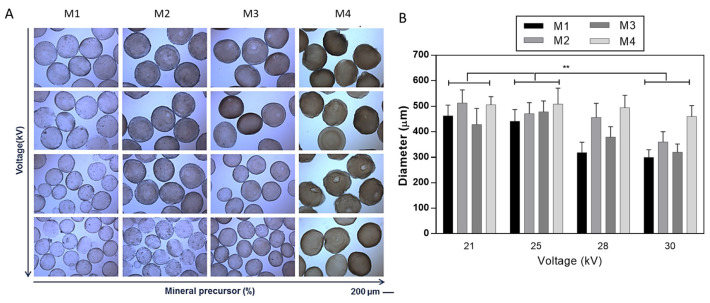
Effect of applied voltage on the morphology of mineralized microgels as a function of the concentration of mineral precursor. (**A**) Optical images (scale bar 200 μm) and (**B**) evaluation of the average diameter via image analysis. Results were presented as mean ± standard deviation (** *p* < 0.01).

**Figure 2:**
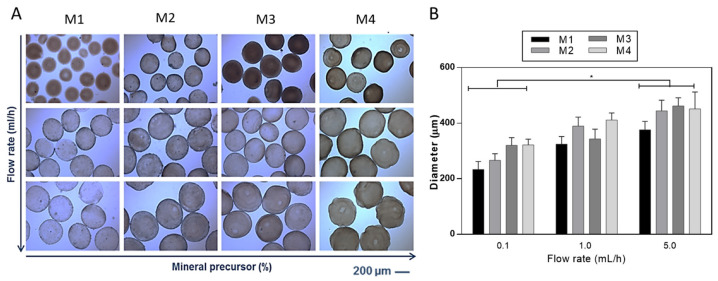
Effect of flow rate on the morphology of mineralized microgels as a function of the concentration of mineral precursor. (**A**) Optical images (scale bar 200 μm) and (**B**) evaluation of the average diameter via image analysis. Results are presented as mean ± standard deviation (* *p* < 0.05).

The development of hydrogel-based scaffolds has been explored in different fields of tissue engineering [26]. To overcome the limitation of alginate-based materials in terms of bioactivity, several chemical and biochemical modifications have been proposed [27,28]. In this work, it was proposed to optimize the fabrication of mineralized microgels triggering the in situ precipitation of mineral phases by adding ionic salts into the SA solution. SA solutions were processed by EHDA, a versatile and efficient technology suitable for the production of narrowly dispersed crosslinked and stable microgels with highly tunable morphological properties [19,29,30]. Despite similar procedures being validated for fabricating composite SA beads in previous works [31,32], the novelty of the proposed method consisted of the use of salts such as disodium phosphates (Na_2_HPO_4_) in the SA solution, acting as precursors for the in situ precipitation of bioactive mineral phases. Herein, it was demonstrated that mineral phases could be efficiently entrapped in the 3D network formed by ionotropic gelation, not limiting the formation of rounded and chemically stable microgels. The presence of mineral phases in the microgels was reported by optical images in Figure 1 and Figure 2, where it is possible to detect a shift from light to dark brown stained microgels as the mineral precursor concentration increases, i.e., 200 mM (M1), 300 mM (M2), 400 mM (M3), and 500 mM (M4).

The effect of process parameters—namely, applied voltage and flow rate—on the morphological properties (i.e., shape, average size, and distribution) was accurately evaluated by optical microscopy supported by image analysis (Figure 1 and Figure 2), and highlighting the contribution of the different salt amount in the formulation used. Figure 1 summarizes the effect of applied voltage on microgel morphology as a function of the mineral precursor concentration (Figure 1). During the atomization process, it is well known that the applied voltage generates a charge accumulation onto the forming droplet surface, which eventually breaks the surface tension, causing the ejection of the droplet [33]. From the image analyses, it was noticed that an increase in the applied voltage tends to promote the formation of smaller microgels due to the faster detachment of droplets from the tip of the needle at a higher voltage that increases the dripping frequency [34]. The influence of applied voltage (from 21 to 30 kV) on microgel diameters was noted by the comparison of samples with the same mineralization content (Figure 1). This effect is significant, especially when the mineral precursor is less concentrated (i.e., M1, M2, and M3), while, for the highest concentration (i.e., M4), the size of microgels did not significantly change.

As expected, it was also verified that the droplet diameter is dependent on the flow rate for a given applied voltage (Figure 2) for samples with the same mineralization content. A significant increase in microgel diameter was detected from the lowest (i.e., 0.1 mL/h) to the highest (i.e., 5.0 mL/h) flow rate values, according to previous works [35], as a consequence of the fluid dynamic mechanism of dripping [36].

**Figure 3:**
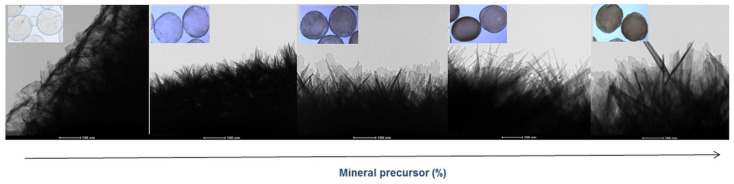
Surface morphology of mineralized microgels as a function of the concentration of mineral precursor via TEM analysis (scale bar 100 nm).

The mineralization of microgels was observed, in detail, by bright-field transmission electron microscopy (TEM) images (Figure 3) of a portion of the microgel surface gels. By comparison with pure SA microgels—which show a smooth surface—it is possible to recognize a diffused presence of mineral crystals with needle-like structures that gradually increase in size as a function of the concentration of mineral precursor used.

The presence of different amounts of the mineral phase in the microgels was also confirmed by thermogravimetric curves that showed higher residue values as a function of the increasing amount of mineral precursor used (Figure S1).

**Figure 4:**
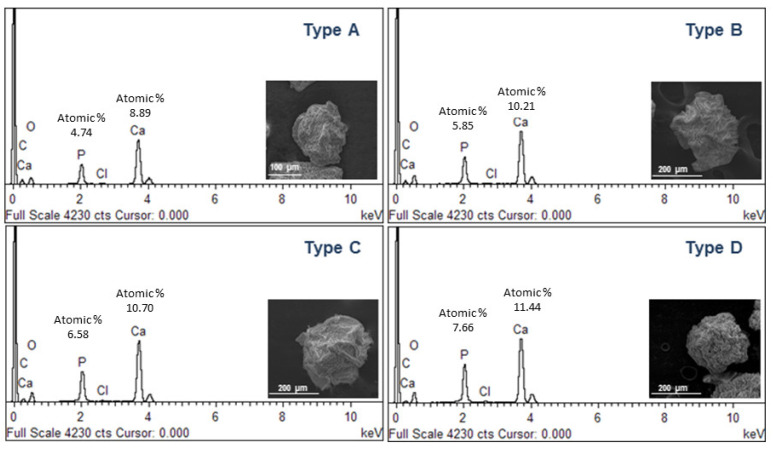
SEM images and EDS analysis of mineralized microgels as a function of the concentration of mineral precursor.

In order to confirm the composition of precipitated crystals, further investigations were performed by scanning electron microscopy (SEM) and X-ray energy dispersive spectroscopy (EDS) (Figure 4). Single particles reported by SEM images qualitatively show a different surface smoothness, ascribable to the different amount of mineral phases formed during the atomization process. Accordingly, the aspect changes, moving from a spherical to a quasi-spherical shape. This can be directly related to the mutual effect of crosslinking and mineralization processes and by the competition of phosphate with Ca^2+^, especially when the system is saturated [37,38]. This leads to the precipitation of mineral phases with a (Ca/P) molar ratio close to the characteristic ratio of stoichiometric hydroxyapatite (Ca/P = 1.67), as confirmed by EDS spectra.

**Figure 5:**
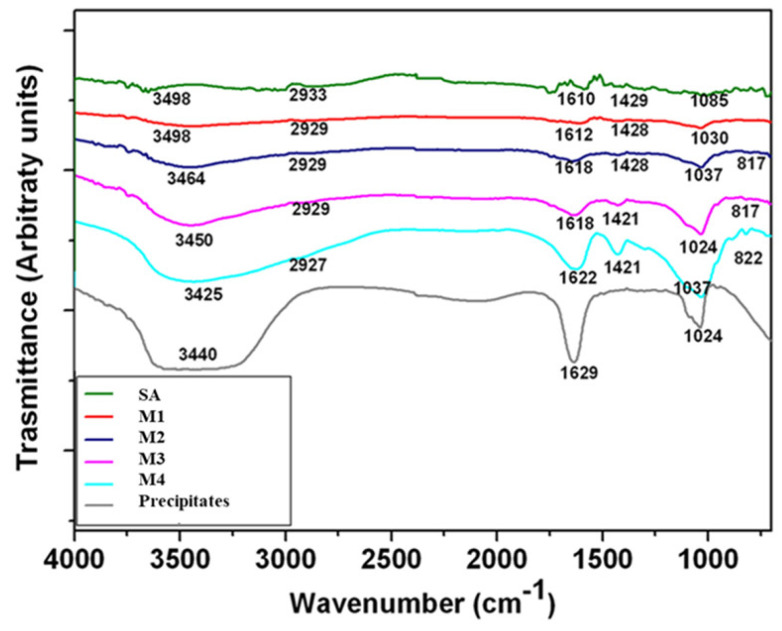
ATR-FTIR spectra of mineralized microgels processed by different mineral precursor concentrations. SA microgels were reported as negative control (SA), while calcium phosphate precipitates obtained by in situ reaction in solution (no atomization) were reported as positive control (precipitates).

Fourier-transform infrared spectroscopy (FTIR) spectra of non-mineralized alginate and mineralized microgels with different concentrations have a similar profile (Figure 5). The FTIR spectra of all microgels showed the characteristic bands of alginate, around 3400 cm^−1^, 2930 cm^−1^, and 1615 cm^−1^, representing the hydroxyl groups, stretching vibration of CH_2_, and symmetric stretching vibrations of -COO hydroxyl groups, respectively [39,40]. The FTIR spectrum of calcium phosphate shows some small peaks between 820 and 880 cm^−1^ and more intense ones around 1460 cm^−1^, formed by CO_3_^−2^ [41]. Moreover, mineralized microgels also showed some bands at 1000 and 1100 cm^−1^, which are characteristic of the PO_4_^−3^ group, which in particular belongs to the hydroxyapatite [42]. The intensity of these bands increased and became sharper as phosphate concentration increased.

Alginate-based materials have been widely used due to their good biocompatibility and good processability to fabricate platforms to work as scaffolds. However, alginate by itself lacks bioactivity to influence cell behavior. CaP is a family that includes several inorganic components of hard tissues (i.e., bone and teeth) that have been used in bone tissue regeneration studies, where its presence promoted cell adhesion, proliferation, and differentiation [43,44,45].

**Figure 6:**
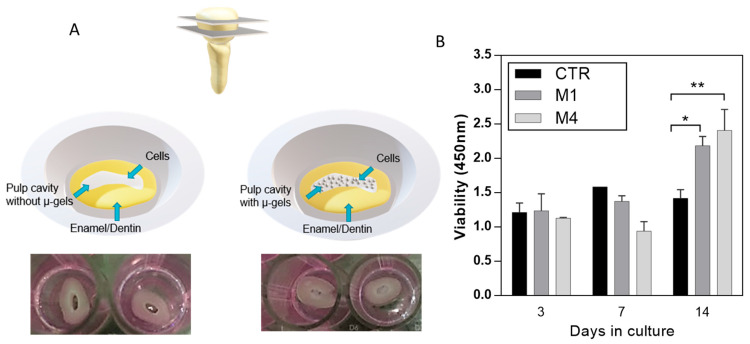
(**A**) Scheme of the preparation of in vitro model of pulp cavity for in vitro assays. (**B**) DPSCs proliferation assays for control (CTR, no microgels) and alginate microgels with low-CaP (Type M1, 200 mM) and high-CaP (Type M4, 500 mM) content. (* *p* < 0.05, ** *p* < 0.01).

For this purpose, the cell proliferation of DPSCs was seeded onto alginate microgels. Low-CaP (M1) and high-CaP (M4) were considered. The in vitro model uses crown sections of natural teeth to mimic the dentin–pulp complex (Figure 6A).

For the experimental samples, microgels were placed in a pulp cavity, and DPSCs were seeded. Cells seeded onto the pulp cavity without microgels were taken as control. In vitro cell proliferation in the control without microgels showed a decreased rate, different from the cells in contact with microgels along the culture time (Figure 6B), due to the high surface area provided by microgels able to support cell proliferation [29]. In addition, CaP is considered an important compound because it is bioactive and provides a suitable substrate for cell attachment and differentiation [46]. Moreover, there is a slightly slow rate of cell proliferation at 7 days, related to the sensitivity of stem cells to the environment and the differentiation pathway at the different stages of cell differentiation, which decreases the metabolic activity [47,48].

In addition, DPSCs are a heterogeneous population of stem cells able to differentiate into odontogenic, myogenic, and adipogenic lineages when cultured in specific media [49,50]. In pathological conditions, DPSCs differentiate into odontoblast-like cells in order to secrete a reparative dentin matrix to form a mineralized barrier to protect the pulp tissue [1].

**Figure 7:**
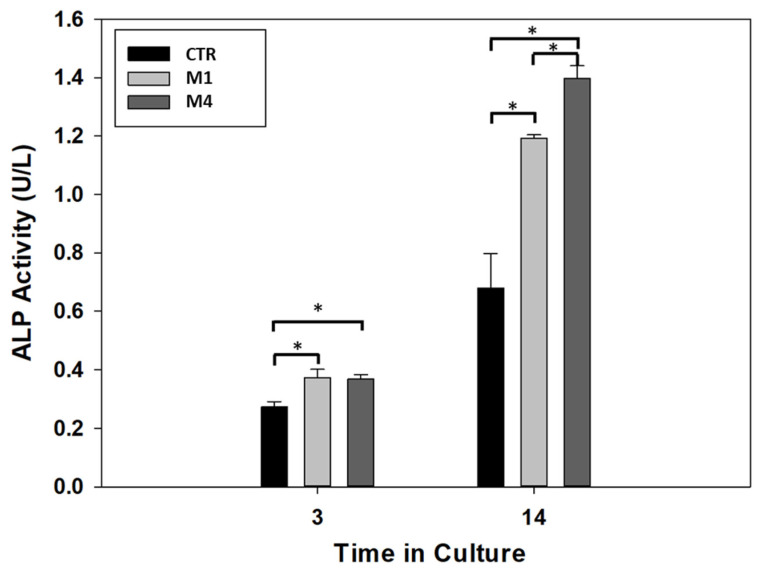
ALP activity of DPSCs in the absence of microgels (CTR) (with lower CaP (Type M1) and higher CaP (Type M2) content). (* *p* < 0.05).

According to recent studies, the differentiation mechanism of DPSC can be addressed by 3D structural cues able to up-regulate some specific bone/odontogenic related genes [51,52] and by chemical cues (i.e., bioactive phases) able to guide osteogenic/odontogenic cell fate and promote the mineral deposition [53,54,55]. In this view, the differentiation of DPSCs cells was evaluated by the presence of alkaline phosphatase (ALP), an early marker commonly used for osteogenic lineage [56]. All the results are summarized in Figure 7.

After 3 days, the ALP activity showed an increment in the two alginate microgels, low-CaP (Type M1) and high-CaP (Type M4), in comparison with the control group (no microgels). However, there is no statistical difference in the ALP activity when the two microgels are compared on the same day of culture, indicating that both microgels modulate the differentiation in this stage. At 14 days of culture, there is an increment in the ALP activity in all conditions. Moreover, the alginate microgel with high-CaP (Type M4) showed higher ALP activity when compared with the low-CaP (Type M1) and control group with statistical differences. Likewise, similar behavior showed the alginate microgel with low-CaP (Type M1) with high ALP activity in comparison with the control group at 14 days of culture. This level of ALP activity showed the modulation of the DPSCs cells under the presence of microgels composite as filler for the regeneration of the dentin–pulp complex, and these results are in agreement with recent experimental evidence about the promotive role of bioceramics on the DPSCs activities regarding osteogenic behavior [57,58,59,60]. In perspective, further investigations should be carried out to detect selected chemical targets involved in the osteogenic differentiation mechanisms of DPSCs, i.e., growth factors such as BMP-2 or signaling pathways such as Wnt/β-catenin, cytokines.

## 3. Conclusions

This work introduced a novel method to fabricate mineralized gels with a micrometric size scale to be used as an engineered in vitro model for the dentin–pulp complex. The optimization of the atomization process allowed, by a single step, different amounts of bioactive phases with apatite-like composition by controlling the concentration of mineral precursor in solution. It was demonstrated that disodium phosphate (Na_2_HPO_4_) salts could efficiently work as mineral precursors to trigger in situ precipitation of mineral phases by reaction of divalent ions in solution, thus promoting an efficient encapsulation of apatite-like phases as confirmed by the characteristic Ca/P ratio close to 1.67. Independently upon the mineral content, they appear as perfectly rounded particles, with average diameters ranging from 223 ± 18 μm to 502 ± 64 μm as a function of mineral content and process parameters used. The definition of in vitro models based on the use of natural teeth slices from human patients allowed us to validate the in vitro response of dental pulp stem cells (DPSCs).

This opens the route to the fabrication of innovative platforms that are able to mimic the microenvironment for cells in terms of characteristic sizes and composition. Indeed, it has been demonstrated that mineralized microgels not only support basic in vitro cell activities (i.e., adhesion, proliferation) but also contribute to trigger differentiation mechanisms in an osteogenic way, thus resulting in a candidate material for hard dental tissue regeneration.

## 4. Materials and Methods

### 4.1. Preparation of Microgels

**Figure 8:**
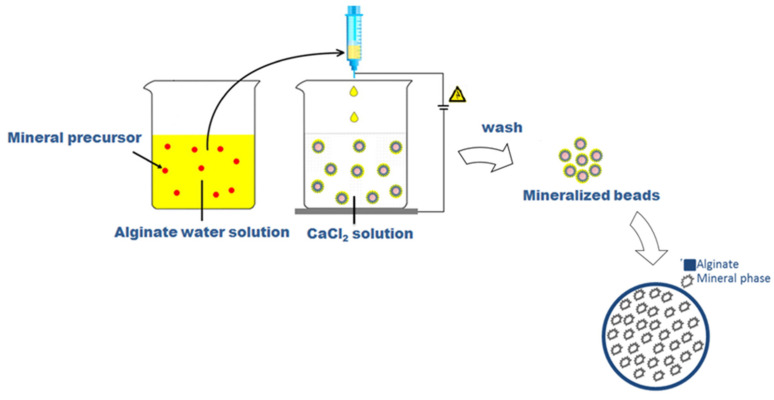
Scheme of preparation of microgels via EHDA.

Sodium alginate (SA) from brown algae (viscosity 20,000–40,000 cPs, Sigma Aldrich, Milan, Italy) was used to prepare a 2% wt/v aqueous solution as a basic formulation to form the µ-gels. Different process parameters, including voltage—i.e., 21, 25, 28, and 30 kV—and flow rate—0.1, 1, and 5 mL/h—were considered during the process, with high reproducibility assured by the use of a commercial machine for pre-industrial use (NF500 MECC, Fukuoka, Japan). In this case, it was equipped with a customized magnetic stirrer acting as a particle collector, in agreement with the patented process [18]. In this work, the native process setup was further adapted to permit the entrapment of mineral phases during the particle formation, as described in Figure 8.

Conventionally, alginate microgels were obtained by collecting SA droplets into a CaCl_2_ (Sigma Aldrich, Milan, Italy) solution under magnetic stirring to trigger the ionotropic gelation of polymers [29]. In the case of mineralized ones, SA was dissolved into a freshly prepared aqueous solution including a mineral precursor, in agreement with similar previous studies [61], i.e., disodium phosphate (Na_2_HPO_4_, Sigma Aldrich, Milan, Italy) with different concentrations: 200 mM (sample M1); 300 mM (sample M2); 400 mM (sample M3); 500 mM (sample M4) in a 2 mL volume, in order to promote the precipitation of calcium phosphates by the in situ reaction with Ca^2+^ ions in the collector bath.

### 4.2. Characterization of Microgels

#### 4.2.1. Morphology

The morphological properties of hydrated microgels were evaluated by optical microscopy (DM750, Leica, Oberkochen, Germany) with the support of image analysis for quantitative measurements of particle diameters. Freeware Image analysis software (Image J, 1.47; NIH, Bethesda, MD, USA) was used for the elaboration of 10 images for each sample group. Results were reported as mean ± standard deviation (SD). Further investigation of microgel morphology was performed via low-vacuum scanning electron microscopy (LV-SEM, Quanta FEG 200 FEI, Eindhoven, The Netherlands), working at low voltage electron emission (<5 kV), to avoid the use of metal coatings of the sample. X-ray energy dispersive spectroscopy (EDAX, Oxford Inca Energy System 250, Wycombe, UK) analysis was used for quantitative estimation of the Ca/P ratio. Lastly, the microgel surface was investigated via bright field transmission electron microscopy (TEM) (FEI Tecnai G12 Spirit Twin, Eindhoven, The Netherlands), equipped with a LaB 6 source and bottom mounted (FEI Eagle 4k CCD camera, Eindhoven, The Netherlands) and side mounted (Olympus SIS MegaView G2 CCD, Münster, Germany) cameras.

#### 4.2.2. Physical and Chemical Characterization

The chemical composition of alginate and composite microgels was examined by attenuated total reflectance technique (ATR-FTIR, Perkin Elmer, Waltham, MA, USA). The spectra were acquired in the region between 4000 and 400 cm^−1^. The analysis was performed using the Origin software (OriginPro 8 SR0; OriginLab Corporation, Northampton, MA, USA). A thermogravimetric analysis (TGA, Q500, TA instrument, New Castle, Denver, USA) of microgels was performed under air from 20 to 600 °C with a heating rate of 2 °C/min, from 25 to 600 °C.

### 4.3. In Vitro Characterization

#### 4.3.1. Isolation and Cell Culture of hDPSCs

To validate the use of microgels as fillers to support the regeneration of dentin–pulp complex, dental pulp cells (DPSCs) were obtained by an explant tissue culture from adult patients who underwent premolar or third molar extraction for orthodontic reasons at the Maxillofacial Surgery Clinic of the Division of Postgraduate Studies and Research at the Faculty of Dentistry of the National Autonomous University of Mexico. (DEPeI-FO, UNAM, Mexico City, Mexico). Patients were informed and signed an informed consent previously approved by the Research and Ethics Committee of the Faculty of Dentistry, UNAM (CIE/1110/2017). After the extraction, the tooth was sectioned with a diamond disk to expose the pulp cavity and extract the dental pulp tissue. Then, the extracted tissue was mechanically disintegrated and incubated with a solution of trypsin 0.25% for 20 min at 37 °C (Sigma-Aldrich, St. Louis, MO, USA) 30 min. Then, fresh culture media was added, and digested tissue was centrifuged at 5000 rpm for 5 min. The supernatant was discarded, and the pellet was re-suspended in fresh media to place in a petri dish for cell culture in alpha-Dulbecco’s Modified Eagle’s Medium (αMEM, Corning, New York, NY, USA) supplemented with 10% fetal bovine serum (FBS, Sigma Aldrich, St. Louis, MO, USA), 2 mM L-glutamine, 10,000 U/mL of penicillin, 10,000 µg/mL of streptomycin, and 25 µg/mL of Fungizone (Gibco Life Technologies, New York, NY, USA) at 37 °C, in a humidified atmosphere with 5% CO_2_. After 48 h, the medium was removed and washed with PBS to remove the unattached cells. Afterward, the culture media was changed every two days, and when cells were in confluence (about 80–90%), they were trypsinized and passed to a 75 cm^2^ flask. For this study, cells from 4 to 6 passages were used.

#### 4.3.2. Preparation of In Vitro Model

Healthy and fresh single-root premolars were stored in a saline solution containing 100 U/mL penicillin, 100 mg/mL streptomycin, and 0.25 mg/mL amphotericin B (Sigma-Aldrich, St. Louis, MO, USA). To mimic the dentin–pulp cavity, cross-sections of the teeth crown were cut (≈1–2 mm) using a diamond disk under constant irrigation.

The crown sections were washed with sodium dodecyl sulfate solution (SDS, Sigma-Aldrich, St. Louis, MO, USA) to eliminate the organic detritus from specimens and then sterilized under ultraviolet light (UV) for 15 min. To fix the cross-section for in vitro studies, the 48-well cell culture plate was coated with a thin layer of agarose. A total of 2 g agarose low gelling temperature was dissolved in 100 mL of Dulbecco’s Modified Eaglemedium (DMEM, Corning, New York, NY, USA). Then, 20 µL of agarose/medium solution was placed into each well to form a thin layer of agarose, and before the complete gasification, the crown sections were placed without filling the pulp cavity.

#### 4.3.3. Cell Proliferation Assay

Cell proliferation was assessed by using a cell counting kit-8 (CCK-8, Dojindo, Kumamoto, Japan) to analyze DPSCs proliferation at 3, 7, and 14 days. CCK-8 is based on the reduction of [2-(2-methoxy-4-nitrophenyl)-3-(4-nitrophenyl)-5-(2,4-disulfophenyl)-2H-tetrazolium, monosodium salt] by dehydrogenases of cells to a water-soluble formazan. For each experimental time, culture media was changed, and 10% (*v*/*v*) of CCK-8 reagent was added to incubate for 4 h. After the prescribed time, the supernatant was recovered and placed into a microplate reader. Absorbance measurements were recorded at 450 nm in a spectrophotometer (ChroMate, Awareness Technology, Palm City, FL, USA). Results were represented as mean ± standard deviation and analyzed by the Student *t*-tests to determine the differences among the groups, considering *p* < 0.05 as statistical significance.

#### 4.3.4. Differentiation Assay (ALP Assay)

The evaluation of the differentiation induction of DPSCs culture with the composite microgels fillers was examined by alkaline phosphatase (ALP) activity after 3 and 14 days in culture. The ALP activity was evaluated by using a colorimetric assay kit (ab83369 Abcamp, Cambridge, UK). Previously, the cross-sections were washed with PBS, transferred to a 1.5 mL microfuge tube for incubation with 250 μL of lysis buffer (Lysis Buffer R2, Gibco Life Technologies, New York, NY, USA), and frozen at −80 °C until use. The ALP activity was performed according to the manufacturer’s protocol. A total of 80 μL of the lysate was added to a 96-well plate with 50 μL of 5 mM of pNPP solution and incubated for the reaction activity at 25 °C for 60 min, and after the period time of incubation, the reaction was stopped with 20 μL of stop solution. The ALP activity was read at 405 nm, and the measurement of the activity in the samples was calculated using a standard curve. The activity was represented as mean ± standard deviation and analyzed by the Student *t*-test to determine the differences considering a *p* < 0.05 value as statistical significance.

## Data Availability

Data are contained in the article.

## Supplementary Materials

The following supporting information can be downloaded at https://www.mdpi.com/article/10.3390/gels9110846/s1, Figure S1: Thermogravimetric analysis of mineralized microgels as a function of the concentration of mineral precursor; Table S1: Mean diameter (µm) of microgels in function of applied voltage. Results are presented as mean ± standard deviation; Table S2: Mean diameter (µm) of microgels in function of flow rate. Results are presented as mean ± standard deviation.
