# Supplementary material for: Mineralized Microgels via Electrohydrodynamic Atomization: Optimization and In Vitro Model for Dentin–Pulp Complex
Source: Gels. 2023 Oct 25;9(11):846. doi: 10.3390/gels9110846 (PMC10670945; doi:10.3390/gels9110846)
Supplement: Supplementary file 1 [file gels-09-00846-s001.zip › gels-2667584-supplementary.pdf]

Supplementary Materials

Mineralized Microgels Via Electro Fluid Dynamic Atomization: Optimization and In Vitro model for Dentin-Pulp Complex

Iriczalli Cruz-Maya 1,2, Rosaria Altobelli 1, Marco Alvarez-Perez 2 and Vincenzo Guarino 1*

1 Institute of Polymers, Composites and Biomaterials (IPCB), National Research Council of Italy, Mostra d'Oltremare Pad.20, Viale J.F. Kennedy 54, 80125 Naples, Italy
2 Tissue Bioengineering Laboratory of DEPeI-FO, Universidad Nacional Autonoma de Mexico (UNAM), Mexico City 04510, Mexico

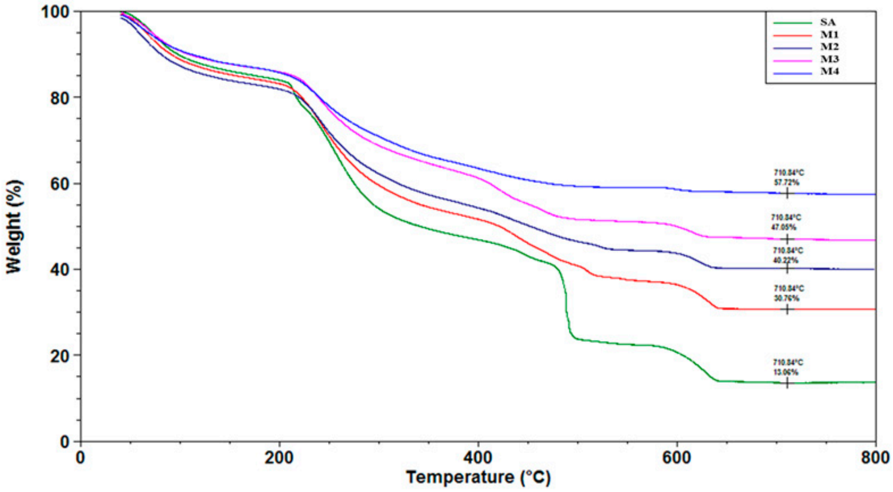

Figure S1: Thermogravimetric analysis of mineralized microgels as a function of the concentration of mineral precursor. An increase of the residue – evaluated after 700°C – indicate an increase of inorganic phase from 30.76% to 57.62% as a function of the increasing amount of the mineral precursor used. Noteworthly, the reported percentage are not exactly the effective amount of the mineral phase, but include other compounds – i.e., salts formed during the process that are stable around 700°C, quantified by the curve of SA sample (i.e., 13.06%).

Table S1. Mean diameter (µm) of microgels in function of applied voltage. Results are presented as mean ± standard deviation.

Applied Voltage (kV)	M1	M2	M3	M4
21	462.3 ± 42.17	512.4 ± 51.3	428.3 ± 63.3	505.7 ± 31.9
25	441.0 ± 45.9	470.7 ± 43.0	478.0 ± 42.6	508.1 ± 62.5
28	317.8 ±40.8	455.7 ± 55.5	378.8 ± 41.3	493.7 ± 49.3
30	299.4 ± 29.93	360.1 ± 39.7	319.5 ± 32.3	459.7 ± 42.6

Table S2. Mean diameter (µm) of microgels in function of flow rate. Results are presented as mean ± standard deviation.

Flow rate (mL/h	M1	M2	M3	M4
0.1	233.6 ± 28.6	266.04 ± 23.9	320.6 ± 27.3	321.95 ± 20.4
1.0	324.3 ± 27.8	389.53 ± 32.4	342.8 ± 35.7	411.6 ± 25.3
5.0	375.7 ± 30.8	444.20 ± 38.5	461.7 ± 29.5	451.4 ±60.7
